# An Intersectional Perspective on Access to HIV-Related Healthcare for Transgender Women

**DOI:** 10.1089/trgh.2016.0018

**Published:** 2016-07-01

**Authors:** Ashley Lacombe-Duncan

**Affiliations:** Factor-Inwentash Faculty of Social Work, University of Toronto, Toronto, Ontario, Canada.

**Keywords:** access-to-care, health disparities, HIV/AIDS, intersectionality, transgender women

## Abstract

Transgender women experience decreased access to HIV-related healthcare relative to cisgender people, in part due to pervasive transphobia in healthcare. This perspective describes intersectionality as a salient theoretical approach to understanding this disparity, moving beyond transphobia to explore how intersecting systems of oppression, including cisnormativity, sexism/transmisogyny, classism, racism, and HIV-related, gender nonconformity, substance use, and sex work stigma influence HIV-related healthcare access for transgender women living with HIV. This perspective concludes with a discussion of how intersectionality-informed studies can be enhanced through studying underexplored intersections and bringing attention to women's resiliency and empowerment.

## Introduction

Systemic exclusion of transgender women^1^ and particularly transgender women of color^2^ is associated with disproportionately high rates of HIV for this population. Access to HIV-related healthcare—including linkage to and retention in care, and initiation of and adherence to antiretroviral (ARV) treatments^3^—is of pivotal importance given that women living with HIV (WLWH) who are not retained in care may not initiate or adhere to ARV treatments, leading to virological failure and death.^4^

Among the small yet growing body of research that focuses on understanding HIV-related healthcare access for transgender WLWH,^5–16^ quantitative studies suggest that transgender WLWH experience differential access,^7,12,16^ including lower retention in care,^16^ current ARV use,^7^ and ARV adherence.^12^ Although most quantitative analyses of HIV among transgender women do not explicitly apply theory, several qualitative studies have employed an intersectional approach to understanding the experiences of transgender WLWH.^5,6,13^

### HIV-related healthcare access disparities for transgender women: intersectionality

Intersectionality is a critical social theory that allows for an understanding of how multiple social identities (e.g., HIV status and gender identity) intersect at the microlevel of transgender WLWH's individual experiences to enact systems of privilege and oppression (e.g., HIV-related stigma and cisnormativity) operating at the macrolevel of society (see Table 1, key definitions).^17–19^ Studies employing an intersectional approach to understanding HIV-related healthcare access for transgender WLWH engage with themes related to macrolevel oppression (e.g., HIV-related stigma and cisnormativity), how these concepts relate to the organization of care (e.g., exclusion of transgender WLWH's needs from healthcare research), and how this translates into the everyday experience of transgender WLWH in healthcare settings (e.g., stigmatizing care).

**Table 1.** **Intersectionality and Transgender Women Living with HIV: Key Definitions**

Term	Definition
Oppression	The systematic and ongoing denial of one group's access to the resources of society by another group (p. 6).^17^
Internalized HIV-related stigma	The degree to which people living with HIV endorse negative beliefs and feelings driven by society about themselves (p. 1163).^21^
Enacted HIV-related stigma	Subtle or overt forms of discrimination as a result of one's HIV status (p. 1163).^21^
Anticipated HIV-related stigma	The degree to which people living with HIV believe they will experience prejudice and discrimination from others in the future (p. 1163).^21^
Cisnormativity	The sociocultural assumptions and expectations that all people are cis-sexual and/or have a cisgender body (p. 356).^33^
Sexism	The belief in the inherent superiority of one sex and thereby the right to dominance (p. 45).^19^
Transmisogyny	The ridiculing or dismissal of a transgender person not merely for failing to live up to gender norms, but for their expressions of femaleness or femininity (p. 14).^28^
Gender nonconformity stigma	Stigma or discrimination of cisgender and transgender women who have a masculine/masculine-of-center gender presentation.^34^
Racism	The belief in the inherent superiority of one race and thereby the right to dominance (p. 45).^19^

Beyond those explicitly applying intersectionality, qualitative studies of access to HIV-related healthcare for transgender WLWH highlight experiences of transphobic stigma and discrimination within and across healthcare settings for transgender WLWH as a particularly salient barrier to HIV-related healthcare access.^5,6,8,10,11,13,15^ Scholars have suggested that prioritizing one category of identity over others does not fully consider the context of inequity in which poor health outcomes proliferate. Thus, these studies also describe how access to HIV-related healthcare is differentially experienced at the intersection of multiple forms of stigma beyond transphobia, including, for example, HIV-related and gender nonconformity stigma.

An intersectional perspective has the potential to enhance our understanding of processes of social exclusion operating across several intersecting oppressions, ultimately informing how we understand HIV-related healthcare access disparities for transgender women. This perspective reviews the literature about access to HIV-related healthcare for transgender WLWH through an intersectional lens. Next, this perspective discusses how intersectionality-informed studies can be enhanced through studying underexplored intersections and bringing attention to women's resiliency and empowerment.

## Applying Intersectionality to Understand Access to HIV-Related Healthcare for Transgender Women

**FIG. 1.**
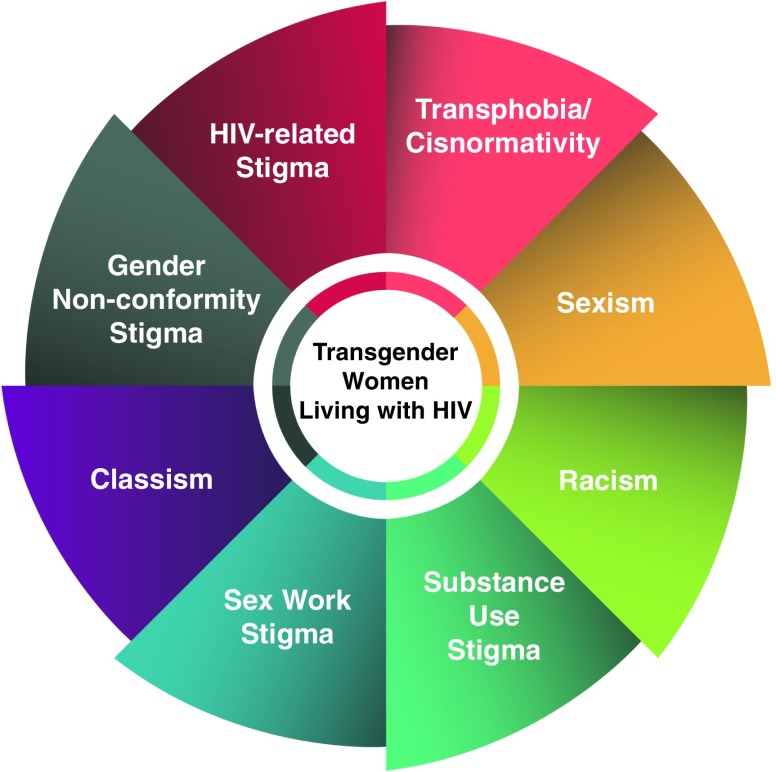
Intersecting stigmas influencing access to HIV-related healthcare for transgender women.

Most research that explores HIV-related healthcare has looked at the experiences of transgender WLWH grouped with women,^5^ LGBT people broadly,^6^ or men who have sex with men.^20^ When studies do focus specifically on transgender women or deaggregate data by transgender identity, the voices of transgender WLWH show a complexity of experiences based on intersecting systems of oppression, including HIV-related stigma, cisnormativity, sexism/transmisogyny, gender nonconformity stigma, classism, sex work, and substance use stigma, and racism (Fig. 1).

### HIV-related stigma

HIV-related stigma is a well-documented barrier to HIV prevention, treatment, care, and support for all people living with HIV.^21^ The impact of HIV-related stigma at a systemic level is evident in research about transgender WLWH, whereby the vast majority of the limited number of studies about transgender women and HIV seek to identify, label, quantify, and reduce HIV risk, while there has been little focus on their treatment, care, and support needs postdiagnosis.^22^ This observation is consistent with broader knowledge production about HIV and women, which tends also to focus on prevention.^23^ Although prevention is essential, this focus on women as “risky” may perpetuate long-held societal stereotypes of WLWH as “diseased” or “dirty,”^24^ and is particularly detrimental toward transgender WLWH who may be subject to additional assumptions (e.g., presumption of sex work) by their healthcare providers.^15^ At an individual level, internalized HIV-related stigma is associated with reduced self-esteem and self-worth, and increased depression or suicidality among cisgender and transgender WLWH,^5^ which also influence access. Both internalized and anticipated HIV-related stigma are associated with decreased engagement in HIV-related care among cisgender and transgender WLWH.^25^

### Cisnormativity

On a systemic level, cisnormativity contributes to an underinvestigation of HIV-related care needs for transgender women, such as a lack of information on the interaction between hormones and HIV medications.^6,10,13^ Physician support of women's continuing their hormones is a facilitator of initiating ARV treatment.^8^ Thus, the exclusion of transgender-specific HIV research poses barriers to HIV-related healthcare access at an individual level for transgender women. Studies also consistently suggest that transgender WLWH report fewer positive interactions with their healthcare providers than cisgender WLWH.^12^

Similarly to HIV-related stigma, anticipated and enacted transphobia may prevent women from disclosing their transgender identity to healthcare providers—limiting their access to gender-affirming care—and may result in avoidance of healthcare altogether.^26,27^

### Sexism/transmisogyny

Serano^28^ describes the intersection between transphobia and sexism as transmisogyny. Transmisogyny has been underexplored in the academic literature to date, and has predominantly been applied in the field of feminist studies to explain the exclusion of transgender women from women's spaces^29^ or to describe interlocking forms of gender oppression.^30^ However, several important links between transmisogyny and barriers to access to care can be made, such as the potential impact of transmisogyny on women's self-esteem or women's safety. For example, transmisogyny may help to explain increased violence against transgender women who do sex work compared with cisgender women.^11^

### Gender nonconformity stigma

Healthcare provider insensitivity, hostility, and ignorance of transgender health are associated with not “passing” as one's identified gender.^27^ Despite the potential importance of gender conformity in promoting HIV-related healthcare access, gender-affirming surgery may be more difficult to access for transgender WLWH than transgender women not living with HIV.^10^ Lastly, gender nonconforming or “not passing” transgender WLWH report increased violence^15^; thus, access to gender-affirming healthcare may play a pivotal role in women's safety and, ultimately, in their ability to access to HIV-related healthcare.

### Classism

Gender nonconformity stigma is often a class-based issue for transgender WLWH—without health insurance, women may not be able to afford gender-affirming care, cannot express their gender, and experience increased risk of violence or discrimination.^8,15^ Moreover, among transgender people, economic discrimination itself is a strong predictor of experiencing violence.^31^ Although poverty is a large issue among WLWH generally, transgender WLWH are more likely than cisgender people living with HIV to experience socioeconomic marginalization on multiple fronts, such as lower income, homelessness, and lack of health insurance,^7,9^ posing a socioeconomic disadvantage to HIV-related healthcare access.

### Sex work and substance use stigma

Sex work stigma, HIV-related stigma, and drug use stigma intersect to limit women's access to HIV-related care.^5,11^ Transgender WLWH are more likely to have a good relationship with their primary care physician if not using substances or involved in sex work.^11^ These intersections are found to contribute to avoidance of care, and subsequent injection drug-related complications such as hepatitis C among transgender WLWH.^11^

### Racism

Few studies have specifically looked at the impact of racism on access to HIV-related care for transgender WLWH. It has been suggested through qualitative work that transgender WLWH of color report a greater sense of fatalism around HIV than white women,^13^ whereas another qualitative study found that racism permeated both individual interactions with healthcare providers and systemic exclusion from research and healthcare planning.^5^ These findings can be contextualized within research among transgender people broadly, which suggests the intersection between cisnormativity/transphobia and systemic racism in producing differential experiences with respect to healthcare access for transgender people of color, such as access to a physician.^32^

## Intersectional Considerations Moving Forward

As demonstrated, an intersectional analysis brings to light the experiences of transgender women situated within multiple intersecting oppressions. Future intersectionality-informed studies can be enhanced by focusing on underexplored intersections and highlighting women's resiliency and empowerment in HIV-related healthcare navigation.

### Underexplored intersections

Few studies examine the HIV-related healthcare experiences of sexual minority transgender WLWH—women who have sex with other women or identify as lesbian, bisexual, gay, or queer. In addition, no identifiable studies discuss the experiences of transgender WLWH with disabilities. Migration and immigration status may also influence women's access to care, yet is often overlooked in studies of transgender WLWH. It is likely that transgender WLWH who have immigrated recently or are refugees have limited access not only to HIV-related healthcare but also to gender-affirming care, a major facilitator of HIV-related healthcare access. Futures studies conducted from an intersectional lens could aim to include sexual minority, differently-abled, and immigrant transgender WLWH.

### Resilience and empowerment

Much research depicts transgender WLWH as a uniformly disadvantaged group where women must struggle to just survive intersecting stigmas. For example, when studies talk about coping, they often talk about “poor” coping,^10^ or the use of substances to cope with stigma.^13^ This depiction overlooks women's adaptive coping mechanisms, resilience, and empowerment. Notably, Logie et al.^5^ discuss women's resilience, resistance to internalized stigma, and self-determination. The authors also highlight how women cope through faith and prayer, joining support groups, and challenging stigma through advocating, political mobilization, and education.^5^ Political mobilization is particularly salient to transgender populations. Barriers and facilitators to healthcare access are often noted as in the hands of healthcare providers or those who organize healthcare. Future studies could further explore adaptive coping mechanisms and highlight women's resiliency and document involvement of transgender WLWH in activism, as well as how this involvement contributes to intrapersonal (e.g., mental well-being) and interpersonal (e.g., HIV-related healthcare provider interaction) factors.

## Conclusions

HIV is a social epidemic that disproportionately affects people experiencing social exclusion across multiple domains of life and based on multiple facets of identity. By applying an intersectional approach to research about HIV-related healthcare access for transgender WLWH, we may develop ways to reduce intersectional stigmatization, build on strengths, increase access, and, ultimately, improve the health and well-being of an over-represented and underresearched population.

## Abbreviations Used

ARV: antiretroviral
WLWH: women living with HIV
